# *Speyeria* (Lepidoptera: Nymphalidae) Conservation

**DOI:** 10.3390/insects8020045

**Published:** 2017-04-25

**Authors:** Steven R. Sims

**Affiliations:** Blue Imago LLC 1973 Rule Ave., Maryland Heights, MO 63043, USA; steve.sims@blueimago.com

**Keywords:** *Speyeria*, conservation, habitats, life history, violets, populations, climate

## Abstract

*Speyeria* (Nymphalidae) are a conspicuous component of the North American butterfly fauna. There are approximately 16 species and >100 associated subspecies (or geographical variants). *Speyeria* are univoltine, occupy a wide range of habitats, overwinter as first instar larvae, and feed only on native violets. *Speyeria* species have become a model group for studies of evolution, speciation, and conservation. Several species and subspecies are threatened or endangered. The reasons for this vary with the taxa involved, but always involve the degradation or loss of quality habitat for larvae and adults. The impacts of climate change must be considered among the causes for habitat degradation and in the establishment of conservation measures. In addition to increasing the available habitat, conservation efforts should consider maintaining habitat in a seral “disturbed” successional stage that selectively favors the growth of violets and preferred adult nectar sources. A major future challenge will be determining the most effective allocation of conservation resources to those species and subspecies that have the greatest potential to respond favorably to these efforts.

## 1. *Speyeria* Biology

*Speyeria* spp. (Nymphalidae) are found throughout most of North America, except for the southeast. There are at least 16 *Speyeria* species in North America and >100 named subspecies [1,2]. All species have a single generation per year (univoltine) and exclusively utilize native violets (*Viola* spp.) as their larval host plants [3]. Females typically do not oviposit directly on the violet host plants but on other vegetation or materials near violets at ground level. After emergence from the egg, first instar larvae seek shelter and undergo an obligate diapause. The diapause larvae are very small (1 to 2.5 mm) and they do not feed until the following spring. Feeding and completion of larval development is synchronized with *Viola* regrowth. The probability of individual larvae surviving through diapause to successfully complete development the following year is certainly very low and *Speyeria* life history strategies reflect selection for reproductive patterns and larval adaptations that maximize larval survival and overall reproductive success [4,5,6].

## 2. Ranges and Habitat Specificity

*Speyeria* inhabit a diverse variety of habitats ranging from high altitude (>3000 m) meadows to low elevations in coastal and interior areas. An example of a high altitude species is *Speyeria mormonia*, which occurs in, and around, alpine meadows generally >2000 m [7,8]. Low elevation taxa include some western California subspecies of *Speyeria callippe* and Oregon and California coastal subspecies of *Speyeria zerene*. Western *Speyeria* species are typically found in mountainous or hilly terrain, where some taxa are associated with wet meadow habitats, while others occur in seasonally dry locations. The range of habitats occupied by subspecies can easily exceed the habitat range of those species that lack subspecies differentiation. For example, *S. zerene*, with 16 described subspecies, occurs in a wide variety of habitats, including coastal dunes and meadows, conifer forests, and *Sagebrush steppe*. *S. callippe* (with >15 described subspecies) occurs in a variety of habitat types, including grasslands, oak and pine woodlands, sagebrush, chaparral, valleys, brushy hillsides, and prairie ridges [2]. In contrast, neither *Speyeria diana* nor *Speyeria edwardsii* have described subspecies [2]. *S. diana* occurs in the Upper Austral to Transition Zone in deciduous and pine woodlands and *S. edwardsii* is found in native prairies, foothills, and meadows.

## 3. Examples of Threatened Species/Subspecies

Examples of threatened species and subspecies of special concern will help to illustrate the range of habitats occupied and the diversity of conservation challenges presented by *Speyeria*. Each of the following taxa has a preferred habitat and the conditions threatening each are different.

### 3.1. Speyeria callippe callippe

*S. callippe callippe* was once widely distributed in the San Francisco Bay area of California, but it is now restricted to a few widely separated populations [9,10]. Its habitat is native grassland, where females oviposit near *Viola pedunculata*. Most of the *S. callippe callippe* decline is due to habitat loss caused by development of the hilly areas of the San Francisco Bay region [11]. Remaining open areas in the region are often disturbed by livestock grazing, recreational use, and invasive non-native vegetation such as grasses and thistles [12]. The San Bruno Mountain *S. callippe callippe* population occurs on land that is mostly protected from development. This area is also being managed for the conservation of several other endangered species, including the San Bruno elfin (*Callophrys mossii bayensis*) and the Bay checkerspot (*Euphydryas editha bayensis*) [13]. The Alameda and Solano County populations are small, but some of the habitat is being protected by state and local agencies [12]. One protected area in Alameda County is located adjacent to a golf course and appropriately named the Callippe Preserve Golf Course (www.playcallippe.com).

### 3.2. Speyeria diana

The main distribution of the Diana fritillary is in the southern Appalachians from central Virginia and West Virginia through the mountains to northern Georgia and Alabama. It is also found in the Ozark Mountains of Arkansas and eastern Oklahoma [3,14]. Populations of *S. diana* are widely scattered within this range and they can have significant interyear variation. *S. diana* is susceptible to habitat changes. Historically, it was more widespread and likely more abundant within its range. Habitat loss in the 19th and 20th centuries reduced its overall range and eradicated *S. diana* from the Ohio Valley, eastern Virginia, and eastern North Carolina. Records for Indiana, Maryland, Ohio, and Pennsylvania date from the 19th century or earlier [15]. Physical threats to *S. diana* are mainly from activities such as logging, residential development, and pesticide applications. For example, use of the selective microbial pesticide *Bacillus thuringiensis* to control pests such as the gypsy moth may cause significant *S. diana* larval kill [16]. *S. diana* can recolonize timbered areas following forest regrowth, but extreme disruptions, such as strip mining, permanently destroy species habitat [17]. The spread of invasive plants may compete with violet host plants and large deer populations may overexploit and reduce violet abundance [18,19]. Populations require substantial expanses of both adult and larval habitat, and these may occur in distinctly separate areas. Adults must be able to locate both habitat types and move between them. *S. diana* may require a larger and more diverse overall habitat than other *Speyeria* species. 

### 3.3. Speyeria idalia

The regal fritillary, *Speyeria idalia,* is found in local scattered populations throughout its range in the Great Plains states from eastern Montana east across the northern U.S. to Maine [2,3]. It is restricted to xeric tallgrass prairie remnants and it is rare or absent from former range east of the Appalachians. There are many extant populations, but the species has suffered severe population declines and its range continues to contract [20] *S. idalia* is almost gone from areas east of the Mississippi River, and it is imperiled in Illinois, Indiana, Pennsylvania, Virginia, and Wisconsin [20,21]. The biggest obvious threat to *S. idalia* is loss of its prairie habitat to development and agriculture, and the principal conservation need is to protect and manage its remaining prairie habitat [22]. In addition to development or conversion of grasslands to agriculture, the remaining prairie has been exposed to pesticides and to fires resulting from prescribed burning. Fires are often, but not always, detrimental to *S. idalia* populations, with recent evidence suggesting that small-scale burning over moderate intervals (3 to 5 years) can benefit populations [23,24].

### 3.4. Speyeria zerene Subspecies

Coastal California and Oregon subspecies of *S. zerene* are especially at risk of extinction [25]. Three of these subspecies are briefly described here. Their life history information and threats are similar, so they will be covered as a group. These subspecies are: Myrtle’s silverspot, *S. zerene myrtleae* [26], Behren’s silverspot, *S. zerene behrensii* [27], and the Oregon silverspot, *S. zerene hippolyta* [28,29]. *S. zerene myrtleae* was formerly widespread on the San Francisco and Marin peninsulas, but is now only known from populations in northwestern Marin County and southwestern Sonoma County. The historic range of *S. zerene behrensii* extended along the northern coast of California, from Sonoma County northward to Mendocino County. It now occurs in a single population at Point Arena. *S. zerene hippolyta* was once common in Pacific coastal meadows ranging from Northern California to Southern Washington. It is now found at only a few sites scattered along the coast of northern California and Oregon. The climate in habitats of the three *S. zerene* subspecies is similar: cool coastal with a long wet season and a relatively short, dry summer. The life histories of the three are similar. The flight seasons range from late June to September and adults feed on nectar from a variety of available flowers. The subspecies are single-brooded and lay their eggs in the dried debris near the larval host plants (typically *Viola adunca*). The first instar larvae overwinter in protected niches and begin feeding on violet foliage in the spring. Major threats to these *S. zerene* subspecies include habitat losses due to human development and vegetation changes [28,29]. The region in which these populations are found is under high pressure for both residential and commercial development. Development has already eliminated many populations and threatens some of the remaining populations. Changes in natural fire patterns, introduction of exotic plants, successional changes in the plant community, grazing, and disruptive recreational activities have also reduced the availability of host plants. The coastal meadow habitats historically used by *S. zerene hippolyta* were maintained in a successional state by periodic fires, wind, and salt-spray, providing the open conditions required by the *Viola* host plants [29]. Wind and salt-spray still limit vegetation growth, but fires have been suppressed because of their dangers to urban structures. Fire suppression is a problem, since *Viola adunca* is better able to germinate and grow when the overlying debris has been cleared by periodic fires; fire also reduces competition from other plants for light and space [30]. With the lack of periodic fires, plant succession occurs and the open meadow habitat is gradually replaced by shrubs and then forest. Invasive exotics such as European beach grass (*Ammophila* sp.) [31], Gorse (*Ulex europaeus*) [32], and iceplant (*Mesembryanthemum* spp.) [26] may limit suitable and timely nectar sources. In addition to habitat conservation, the preservation of the three *S. zerene* subspecies may depend on more active measures. Re-introduction of periodic fires is an obvious first step. Adequate densities of the host plant *Viola adunca* are also important and may need augmentation by supplemental planting efforts [33,34]. Adult nectar sources are critical and must be present in adequate numbers during the adult flight season. This may require selective removal of competing alien plants and the planting of native plant species that are documented nectar sources and known to flower at the appropriate time. Selected non-invasive alien species should also be considered for planting as nectar sources [33].

## 4. Dispersal and Gene Flow

The level of dispersal between isolated *Speyeria* populations is a species-specific parameter that affects both gene flow and the probability of suitable habitat re-colonization. Suitable habitat areas could include both new habitat and areas where previous populations have gone extinct. Dispersal among *Speyeria* populations ranges from relatively low in *Speyeria nokomis* [35] and *S. diana* [36] to relatively high in *S. coronis* [37] and *S. idalia* [34,38]. *S. diana*, *S. idalia*, and *S. nokomis* represent taxa with fragmented populations that are often separated by large distances [15,35,39]. In these groups the potential for inbreeding and loss of genetic diversity (heterozygosity) may be relatively high [40,41]. Loss of genetic diversity can contribute to a reduced ability to adapt to a changing climate and other environmental conditions [42]. Connectivity between discrete populations is important and this can be provided by habitat steppingstones [43], passages of continuous suitable habitat [44], or human-assisted movement [45,46]. Lack of adjoining land-based corridors between habitats does not necessarily impair *Speyeria* movement [47] or conservation, but it could make it more difficult for some species [48]. In other groups of butterflies, inter-patch distance can affect colonization and population persistence [49,50,51] and corridors can enhance movement between habitat patches [52,53]. For example, it is not uncommon for some butterfly species to regularly cross two-lane highways [54] or traverse entire urban landscapes, while for other species, movement across such barriers is limited or absent [55]. Some investigators have proposed that stepping stones of suitable habitat may be more effective than corridors for supporting inter-patch dispersal [43]. Dispersal of habitat specialist species such as *S. nokomis* over inhospitable habitat appears to be rare [35], while other species such as *S. coronis* are considerably more vagile and better able to locate specialized habitats within the matrix of a large area [37]. Therefore, many *Speyeria* conservation strategies could reasonably focus on protection of all suitable habitat areas, even those areas that are very small. This should include potential habitat possessing appropriate food and nectar plants, but located outside of currently occupied habitat.

## 5. Climate Change, Life History Traits, and Habitat Adaptations

In addition to the direct effects on habitat quantity and quality caused by human disturbances, the effects of climate change must also be considered and could indeed be the greatest current threat to *Speyeria* population survival [56,57,58]. For example, relatively common species such as *Speyeria aphrodite* and *Speyeria atlantis* are declining in formerly favorable habitats of Massachusetts as the climate warms, and the northern ranges of many other butterfly species are also being negatively affected [57,58,59]. Climate change not only involves “average” climate changes, but also changes in the frequency and severity of catastrophic weather events [60]. *Speyeria* habitats will become warmer and drier in some locations, but perhaps wetter in others, and there will likely be greater between-year weather variation. Some sequences of years may have record low rainfall and drought (e.g., California 2010 to 2015). Summers in areas such as California are prolonged, compared to historical conditions, with warmer days and longer periods of warm days without rain [61,62]. Elevation and latitude will interact with climate change and changes may be more severe at higher elevations and higher latitudes [63]. The flight duration of late season flying *Speyeria* species may become extended, as it has for some European *Argynnis* [64]. The beginning of the flight season can also occur earlier in the year. For example, the initial flight date for female *S. diana* is now an average of 4 days earlier than it was in the early to mid-20th century [15]. Climate change has already influenced *Speyeria* populations as well as populations of violets, and these changes can affect population performance, especially in marginally suitable environments [65]. *S. diana*, and many other insects, have responded to climate change by gradually moving into habitats located at higher latitudes or elevations [15,57,58,64,66]. However, this option is unavailable for *Speyeria* species such as *S. mormonia*, already surviving at high elevations, or *S. idalia*, for which elevational shifts are not possible.

Changing climate directly effects two important parts of the *Speyeria* life cycle. The first component is female reproductive diapause. Overwintering first instar *Speyeria* larvae are particularly vulnerable to mortality from abiotic stresses such as desiccation [5]. The odds of larval survival over the summer-fall-winter period can be increased through female reproductive diapause, the occurrence of which varies with species and habitat [6]. In species such as *S. coronis* and *S. idalia,* all females have a reproductive diapause. In *S. zerene*, subspecies from low-mid elevation xeric habitats typically have a reproductive diapause, while subspecies from high elevations or coastal areas appear to either lack, or have a reduced, diapause [6,29]. Cessation of reproductive diapause is influenced by photoperiod [67] that delays oviposition until late summer–early fall. Late season oviposition reduces the period during which the first instar larvae are exposed to desiccating conditions and decreases the interval between egg hatch and the beginning of cooler and moister fall weather. The second life cycle adaptation is diapause intensity and desiccation tolerance of first instar larvae. Diapause intensity is the duration of diapause under given (typically favorable) environmental conditions. First instar larval diapause intensity varies significantly among species and between individuals of the same species [5]. Diapause intensity enables first instar larvae to remain in a physiological state that “buffers” them from environmental extremes of heat and drought, and diapause larvae are able to survive drought and desiccation better than non-diapause, metabolically active, larvae. In dry habitats, larvae that prematurely end diapause prior to host plant availability will perish. Climate change and warmer-drier conditions makes synchronizing diapause termination and violet growth more difficult. A longer period of warm weather during diapause depletes the metabolic reserves (fat body, sugars, etc.) that the larva needs to survive during the winter. Climate change could cause diapause to end prematurely before violet foliage is available. First instar larvae resistance to desiccation also varies significantly among species and populations, suggesting that diapause intensity and desiccation tolerance are heritable traits that could be selected upon for population adaptation to changing climates [5,68,69]. Selection pressure from climate changes may result in first instar *Speyeria* larvae with greater tolerance to extended diapause and increased resistance to desiccation. Selection for a longer female reproductive diapause would also reduce the interval of desiccation stress faced by first instar larvae.

## 6. Violet Availability

Violets are essential in *Speyeria* biology, but their population dynamics and taxonomy are poorly known [70,71]. The present and future effects of climate change on violet hosts are also unclear. Within some habitats, climate-induced changes in violet populations are likely. For example, populations of drought tolerant dry habitat violet species might outcompete, or simply replace, species that require more moisture. Alternately, changing climate might reduce violet populations or favor plants that will outcompete the violets. It is difficult to locate and study *Speyeria* larvae on violets under field conditions [72]. The abundance and distribution of violet plants affects the probability that a *Speyeria* larva can locate violet foliage in amounts adequate to complete development. This would be especially evident if the density or distribution pattern of violet populations were changed. In a dispersed violet distribution, partially grown larvae might need to search a greater distance to locate the next violet host. There would be no guarantee that a host would be found and so this could increase larval mortality. In addition, increased search times would expose larvae to greater risks from biological hazards such as predators and parasitoids. Survival chances of a larva would increase if, after complete consumption of the foliage of one violet host plant, it could move away from this plant and readily locate another. Under field conditions, larger, older larvae of *S. zerene hippolyta* moved more rapidly and turned less acutely than smaller, younger larvae [73]. Younger larvae thus tended to remain in one place while older larvae, in search of new host plants, tended to range more widely. A simulation model initialized with these data suggested that a host plant density of four *V. adunca* plants/m^2^ would be required for fourth instar larvae to have at least a 10% chance of survival to pupation [73]. There is no evidence that violet host plants differ in nutritional value for *Speyeria* larvae; the major limitation seems to be adequate plant biomass [74]. Large species, such as *S. diana* and *S. idalia*, require greater amounts of violet foliage for larvae to complete development than small species such as *S. mormonia*. Some studies have found a positive correlation between violet density and population numbers of *Speyeria* [75], but other studies report no obvious correlation [76]. Because *Speyeria* eggs are often not deposited directly on violet hosts, a high density of violets in the immediate area would increase the probability of the first instar larvae reaching a host plant before mortality from starvation.

## 7. Conservation Issues

As is the case for many other endangered or threatened insect species, the most significant problems facing *Speyeria* have involved loss of suitable larval and adult habitats and a reduction in the quality of remaining habitat [77,78]. The majority of habitat losses have resulted from human activities, including land development for agriculture, commercial, and residential uses, but climate change now represents an additional risk factor. In some cases, *Speyeria* habitat has been reduced in a mosaic pattern, leaving relatively small islands of suitable habitat that may be inadequate for long-term population survival [21,26]. In addition to the inbreeding and reduced gene flow problems mentioned previously, small populations also face an increased risk of extinction from stochastic events such as severe weather or fires [79]. If a species lacks sufficient dispersal capability and is unable to re-colonize this habitat from larger metapopulations then local extinctions will contribute to a decline in the overall species range. Certain habitats are at higher risk for disruption because of human activities. These include prairie habitats that are valued for agriculture and livestock production and coastal habitats that are desirable for residential and commercial purposes. As a result, *Speyeria* populations in these habitats have suffered disproportionally. In those areas that have avoided significant disturbance and development, there remains the problem of habitat deterioration or quality loss [59]. A component of this deterioration involves invasive species that are, or could be, detrimental to *Speyeria* survival. Invasive plants can outcompete violet larval host plants and reduce the abundance of adult nectar plants [17]. It is also possible that introduced natural enemies such as predators and parasitoids have had a negative influence on *Speyeria* larvae, but this risk has not been evaluated. Adequate numbers of nectar-producing plants are needed to nourish adults [80]. Reproductive potential is maximized when females are supplied with energy from the sugars provided by flower nectar. Nectar may also provide amino acids that can contribute to adult longevity and fecundity [81]. The flowers providing nectar during *Speyeria* female reproductive diapause change through the summer months, and the late summer flowers may differ in nutritional value compared to those found earlier in the season. Females of *S. diana* and *S. idalia* chose high quality nectar sources which probably enhance both adult survival and fecundity [36,82]. A decline in these nectar sources in the field has not been definitively linked to reduced population success in *Speyeria*, but this has occurred in other butterfly species [83,84]. There have been suggestions that only native plant species can accommodate *Speyeria* nectar requirements, but some introduced species such as *Circium* and *Carduus* spp. are acceptable nectar sources for coastal *S. zerene* subspecies [26,33].

A generic conservation approach will not be effective for all *Speyeria* because of the diversity of habitats these groups occupy. For some species, a “hands off” approach could be the best option. This may be true for the mid to high elevation mountain species, where conservation and preservation of large expanses of preferred habitat is possible and would be the most economical policy to implement. However, montane species are not immune from the effects of human disturbances and climate changes, especially those groups with fragmented populations [85]. For species restricted to wet habitats (e.g., *S. nokomis*), the obvious conservation strategy will be to avoid activities that disturb habitat integrity or promote drying (these adversely affect violet growth). Avoidance of development, drainage, excessive animal grazing, and agrichemical use is desirable [86]. Corridors between suitable, but separated, wet meadow habitat areas could be advantageous.

For species such as *S. diana* and *S. zerene*, a degree of habitat disturbance can not only be tolerated, but is essential if the disturbance enhances larval host plant violet growth and simultaneously increases the abundance of adult nectar sources [30]. Greater numbers of *S. diana* adults are often seen in “ecotonal” areas created by controlled fires or by selective logging [17,87]. These disturbed areas are a successional seral stage that may be more favorable to violet and nectar flower growth. *S. idalia* seems to do best on relict tall grass prairie habitat, so efforts should be made to continue expanding these areas and to provide corridors between separated prairie areas. Coastal *S. zerene* populations should benefit from habitat preservation and management of invasive plant species that alter their preferred semi-open habitat, reduce solar radiation, and compete with violets [25,33].

The examples provided above suggest that for *Speyeria*, as for many other endangered species, preservation of quality habitat is a primary, but not sole, consideration for survival. Also, for butterfly species that persist only on remnant habitats, it is easy to assume that these sites are models of high-quality species habitat. However, populations of endangered *Speyeria* may occur in locations that do not represent, historically, the best conditions for the species (or subspecies). The ideal habitat characteristics and the processes by which they were maintained may not be obvious without detailed knowledge of the biological history of the species outside its remnant distribution.

An important aspect of conservation is determining the necessary and sufficient area of habitat required and the extent to which this habitat area is “entire” in contrast to “fragmented”. *Speyeria* species (and subspecies) are composed of many small populations that, in total, comprise metapopulations. It is important to recognize and focus on the areas with the largest and most consistent populations, as these are indicators of high quality habitats. All suitable habitats must include adequate amounts of both larval (native violets) and adult flower food sources. Some habitats require special intervention efforts to enhance the survival and performance of the violets and nectar sources. These interventions may involve periodic controlled fires or other means to reduce ground litter and eliminate competing alien and native plants. Reduction of alien plants might require manual removal in areas where fires are not permitted. Butterfly conservation in general, and *Speyeria* conservation in particular, recognizes the important role of natural disturbance in protected lands [88,89]. The role of disturbance is essential to healthy functioning of these lands, since rare and endangered butterfly species often depend on larval foodplants that require various habitat disturbances for their long-term persistence and health. Many *Speyeria* habitats no longer experience these natural disturbances and so a common conservation approach of minimizing human disturbances results in gradually degraded habitat for both host plants and nectar sources. Disturbance is very important where food plants are being excluded by exotic species through direct competition. For example, the *Plantago* foodplants used by Bay Checkerspots grow better and butterflies thrive when cows graze annual exotic grasses [90]. Intentional and directed use of natural disturbances will be necessary to maintain habitat for many endangered butterflies [91]. These efforts may come into conflict with management of habitat for later succession species and resistance from land managers or a public accustomed to a hands-off approach to conservation management. Some *Speyeria* habitats may never be self-sustaining and will require periodic maintenance. Lacking detailed studies of optimal conditions, it may only be a guess as to exactly what habitat parameters to manipulate and to what degree. The dependence of many butterfly species on early-successional foodplants means that habitat cannot simply be restored, but must be continually re-created. Current *Speyeria* habitats, even those that appear currently suitable for species survival, may be evolving in the direction of unsuitability due to hard-to-define alteration of interspecific associations. For example, *S. callippe callippe*, within its population on San Bruno Mountain in the San Francisco Bay Area, has historically had greater reproductive success in areas that avoid a westerly (facing the Pacific Ocean) exposure [13], but these areas are being encroached by shrub growth that requires periodic removal.

## 8. Captive Rearing

With increasing numbers of butterfly species facing extinction, captive rearing programs are often considered as a management tool to augment or repopulate threatened populations. Captive breeding of vulnerable butterfly populations has played an important role in the recovery and ongoing conservation of many threatened and endangered species, but the effectiveness of these efforts can be unclear due to inadequate monitoring and follow-up [92]. Nevertheless, some butterfly species appear to have greatly benefitted from population augmentation enabled by captive breeding programs [93]. Augmentation efforts are rare with *Speyeria* because they are difficult to successfully rear in captivity and the rearing process is labor intensive [94,95]. Captive reared *S. zerene hippolyta* have been used in a few conservation plans [96], but little other work has been done. Unless habitat parameters are also improved in the release sites, the value of these efforts may be short-lived [94,97,98].

## 9. Conclusions

From a geological perspective, periods of rapid and extreme environmental changes are common, and we are in the midst of another such period. Ecological change is also the norm, rather than the exception, and communities, with their associated species, come and go. One relevant question we face in dealing with endangered *Speyeria* is deciding on the most beneficial allocation of scarce resources for the preservation of those species (populations) with the greatest potential to benefit from such efforts. Even for the many species and populations that are formally listed as endangered by federal (U.S. Environmental Protection Agency) or state agencies, there may be inadequate time and money to save them. In addition, the genetic distinctiveness of these populations remains in question. Despite reasonably clear morphological differences, most subspecies, and many species, of *Speyeria* are indistinguishable genetically and are capable of producing fertile interspecific hybrids [99]. This seems true for many endangered taxa such as the *Apodemia mormo* (Riodinidae) group and others [100,101,102]. *Speyeria* species and subspecies are likely to be of relatively recent origin, and this should be considered when evaluating genetic data [2]. Using information from multiple genetic markers combined with traditional taxonomic data is probably a more realistic approach to clarify taxonomic questions related to conservation targets [103]. In the near term, it may be misguided to neglect devoting attention and resources toward the conservation of clearly defined *Speyeria* subspecies and populations that are threatened. However, in the long term, it may be more practical to combine scattered conservation resources and focus on those populations with the potential to benefit most from conservation efforts.
